# KEGG spider: interpretation of genomics data in the context of the global gene metabolic network

**DOI:** 10.1186/gb-2008-9-12-r179

**Published:** 2008-12-18

**Authors:** Alexey V Antonov, Sabine Dietmann, Hans W Mewes

**Affiliations:** 1 GSF National Research Centre for Environment and Health, Institute for Bioinformatics, Ingolstädter Landstraße 1, D-85764 Neuherberg, Germany; 2 Department of Genome-Oriented Bioinformatics, Wissenschaftszentrum Weihenstephan, Technische Universität München, 85350 Freising, Germany

## Abstract

KEGG spider is a web-based tool for interpretation of experimentally derived gene lists in order to gain understanding of metabolism variations at a genomic level. KEGG spider implements a 'pathway-free' framework that overcomes a major bottleneck of enrichment analyses: it provides global models uniting genes from different metabolic pathways. Analyzing a number of experimentally derived gene lists, we demonstrate that KEGG spider provides deeper insights into metabolism variations in comparison to existing methods.

## Background

In the post-genomic era the targets of many experimental studies are complex cell disorders [1-6]. A standard experimental strategy is to compare the genetic/proteomics signatures of cells in normal and anomalous states. As a result, a set of genes with differential activity is delivered. In the next step, the interpretation of identified genes in a model context is required. A widely accepted strategy is to infer biological processes that are most relevant to the analyzed gene list. The inference is based on prior knowledge of individual gene properties, such as gene biological functions or interactions. This common approach is usually referred to as enrichment analysis [7-16].

The Kyoto Encyclopedia of Genes and Genomes (KEGG) is a knowledge base for the networks of genes and metabolic compounds. The major component of KEGG is the PATHWAY database, which consists of graphical diagrams of biochemical pathways, including most of the known metabolic pathways. Several available public tools, such as GenMAPP/MAPPfinder [17], PathwayProcessor, and PathwayMiner [18], make use of standard enrichment analysis to find overrepresented global pathways within a gene list. However, for statistical evaluation these tools use only information about gene pathway membership, while information about pathway topology is largely discarded. Additionally, several tools provide visualizations of pathways reported to be enriched [19-21]. Some tools provide visualizations of a gene list in the context of the global metabolic network [22,23], providing, however, no quantitative or statistical analyses. Visual analyses of the graphical representation of the genes on the global metabolic network give only an intuitive feeling that genes are related. Taking into account the density of metabolic networks, one must not underestimate the value of a statistical treatment. Even for randomly generated gene lists, it is possible to connect many of the genes into a metabolic subnetwork through one or two intermediate partners. A graphical representation may have low scientific value without providing a quantitative estimate of the model quality.

More complex statistical methods have been proposed to take pathway topology into account by developing specialized scoring functions. For example, in the ScorePAGE method the distance between genes within the metabolic pathway is included into the scoring function [24]. In this case, the impact of a pair of genes is weighted with respect to the distance between genes within the metabolic pathway. Another recently proposed procedure (impact analyses) [25] exploits the hierarchical structure of signaling pathways and weights the impact of genes with respect to their position in the pathway hierarchy. Genes at the top of the signaling cascade receive higher impact in comparison to downstream genes.

We propose a novel statistical approach for the analysis of gene lists in the context of gene metabolic pathways that uses network topology to make knowledge inference. Our approach does not evaluate each individual KEGG metabolic pathway separately, but uses a global gene metabolic network that integrates all KEGG metabolic pathways together. The input gene list is translated into a network model, e.g. edges connect genes that most probably affect the state of each other. We also proposed a robust statistical treatment of the inferred network. As an output, our procedure provides a graphical model as well as statistical significance of the inferred network computed by a Monte-Carlo simulation procedure. We show on several real data sets that our approach provides deeper insight into variations of metabolic pathways covered by the given gene list in comparison to currently available methods.

## Results and discussion

**Figure 1:**
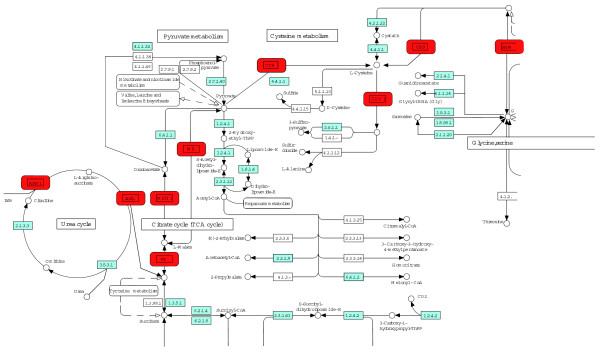
**Artificial example**. The genes *ME1*, *MDH1*, *FH*, *ASL*, *ASS1*, *CTH*, *CDO1*, *CBS *and *SHMT1 *are presented as red boxes. Five KEGG pathway ('urea cycle', 'citrate cycle', 'pyruvate metabolism', 'cysteine metabolism', 'glycine, serine and threonine metabolism') wiring diagrams are manually linked together to demonstrate that all nine genes form a non-interrupted metabolic network.

Let us start from consideration of an illustrative example to highlight the weaknesses of existing analytical methods. Assume that as a result of some experiment one gets a list of nine human genes, *ME1*, *MDH1*, *FH*, *ASL*, *ASS1*, *CTH*, *CDO1*, *CBS*, *SHMT1*. These genes are related to metabolism, and an enrichment analysis would identify several overrepresented metabolic pathways. Three genes (*CTH*, *SHMT1*, *CBS*) are mapped to 'glycine, serine and threonine metabolism'. Two genes (*ASL*, *ASS1*) are mapped to 'urea cycle' and two genes (*ME1*, *MDH1*) are mapped to 'citrate cycle'. No functional model that unites all nine genes together would be supplied by standard enrichment analysis. However, according to the KEGG pathway wiring diagrams shown in Figure 1, all nine genes are consecutively connected via metabolites and form a non-interrupted network that runs through five canonical KEGG metabolic pathways, namely 'urea cycle', 'citrate cycle', 'pyruvate metabolism', 'cysteine metabolism', and 'glycine, serine and threonine metabolism'. This illustrative example demonstrates that, in many cases, the knowledge of enriched pathways may be insufficient to get a complete understanding of the relationship between genes from the supplied list. Consideration of the topology of the global gene metabolic network for the interpretation of gene lists may be much more informative.

We assume that the closer the genes on the global gene metabolic network, the greater the probability that the change in the state of one gene will affect the state of the other. In the considered illustrative example in Figure 1, *ASS1 *and *ASL *are both associated with L-argininosuccinate. Thus, the change in the state of *ASS1 *(for example, overexpression) most probably affects the amount of L-argininosuccinate in the cell (Figure 1). There are probably many ways the cell can handle extra amounts of L-argininosuccinate. One of them is to increase the efficiency of its utilization through possible metabolic reactions. The cell response can be the increased level of *ASL *expression. The *ASL *overexpression will speed up L-argininosuccinate transformation into fumarate and arginine. Thus, even if two genes are not directly involved in regulatory relationships, but catalyze close reactions on the global network, they can affect the state of each other through auto-regulatory mechanisms switched up by abnormal amounts of common metabolites.

### KEGG spider

KEGG spider [26] is a freely available web-based tool that implements a global metabolic network framework for the interpretation of gene lists. It has a simple interface: as input it accepts several types of gene or protein identifiers. For example, for the human genome, KEGG spider supports identifiers from 'Entrez Gene'[27], 'UniProt/Swiss-Prot', 'Gene Symbol' [27,28], 'UniGene' [27], Ensembl' [29], 'RefSeq Protein ID', 'RefSeq Transcript ID' [30], and'Affymetrix probe codes' [31]. As output, the user gets a report on the statistical significance of the inferred network models (*D*_1_, *D*_2_,..), as well as a catalog of enriched KEGG pathways and Gene Ontology terms. For each model (*D*_1_, *D*_2_,..), a link is provided to obtain a graphical visualization. The visualization is performed by the Medusa package [32]. In addition, the user can highlight genes from the model according to KEGG canonical pathways. The inferred network models can be downloaded as a text file and used with freely available packages for network analyses and visualization [32,33].

Here, we present several examples of analysis of published experimental data by KEGG spider. To illustrate the advantages experimental researchers would get by using KEGG spider in comparison to commonly used pathway enrichment analyses, we provide a comparison between KEGG spider and GENECODIS [34], a tool recently published in *Genome Biology *that implements a possibility to perform enrichment analysis of KEGG pathways. The choice of GENECODIS was casual, as the results of enrichment analyses of KEGG pathways by other tools would be similar.

We also provide a comparison (Additional data file 1) of KEGG spider to KEGG atlas [23]. KEGG atlas is a web tool that provides visualization of a gene list (converted into KEGG KO identifiers) in the context of the global metabolic network. As has been discussed above, KEGG atlas provides no quantitative or statistical analyses and, thus, supplies no criteria for the evaluation of the quality of provided graphical output. As demonstrated, the output of KEGG atlas for a random gene list looks similar to the experimentally derived gene lists.

### Identification of genes commonly up- or downregulated in diffuse-type gastric cancers

In [35] a comparison of the expression profiles of cell populations from 20 diffuse-type gastric cancers with their corresponding non-cancerous mucosae was performed. The authors report in the paper the top 75 up- regulated and top 75 down-regulated genes. The 150 differentially expressed genes represent a variety of functions, including genes involved in various metabolic pathways. In total, 28 genes map to KEGG metabolic pathways. Enrichment analysis (Table 1) identified three pathways that are significantly overrepresented. For example, nine genes are from the 'metabolism of xenobiotics by cytochrome P450' pathway and five are involved in 'bile acid biosynthesis'.

**Table 1:** KEGG metabolic pathways enriched in the list of 150 genes (28 genes map to KEGG metabolic pathways) commonly up- or down-regulated in diffuse-type gastric cancers [35] (reported by GENECODIS)

Number of genes	*P*-value (not corrected for multiple testing)	KEGG pathway
9	4.42E-18	(KEGG) Metabolism of xenobiotics by cytochrome P450
5	2.20E-10	(KEGG) Bile acid biosynthesis
5	2.40E-09	(KEGG) Glycolysis/gluconeogenesis

**Figure 2:**
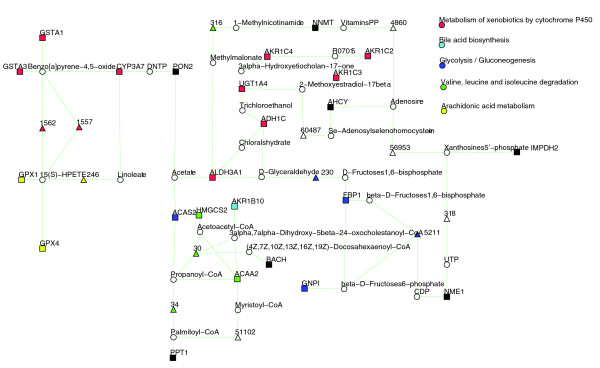
**Network model *D*_2 _of 150 commonly up- or down-regulated genes in diffuse-type gastric cancers **[35]. Twenty-eight genes can be mapped to KEGG metabolic pathways; the model *D*_2 _covers 24 genes (*p*-value < 0.001). Genes from the input list are presented as rectangles, intermediate genes as triangles and chemical compounds as circles. Different colors are used to specify different KEGG canonical pathways.

The model *D*_1_, containing directly connected genes, provided by KEGG spider covers 14 genes (*p*-value < 0.001). The model *D*_2_, in which one intermediate gene is allowed, covers 24 genes (*p*-value < 0.001). Figure 2 presents a graphical visualization of the inferred D2 model, which spreads through five canonical KEGG pathways.

Therefore, in comparison to available analytical procedures, KEGG spider enhances our understanding of metabolism variation in gastric cancers. First, it demonstrates that deregulated genes do not split into independent groups (pathways) as may be concluded from standard enrichment analyses: almost all 24 (out of 28) genes form a non-interrupted (a maximum of one missing gene is allowed) network. Second, it provides not only information that 24 genes are mapped close to each other on the global metabolic network but also estimates the confidence of this event: the *p*-value reflects the probability of getting a non-interruptedly connected network that covers at least the same number of genes for a randomly sampled list of 28 genes (only genes mapped to KEGG metabolic pathways are used to generate the random lists).

### Proteomic analysis of livers of patients with primary hepatolithiasis

Primary hepatolithiasis or intrahepatic calculi, which is characterized by the formation of gallstones in the intrahepatic bile duct, is an intractable liver disease and suspected to be one of the causes of cholangiocellular carcinoma. To obtain an insight into the disease, the proteomic analysis of liver tissue specimens was done (affected and unaffected hepatic segments from patients with primary hepatolithiasis) [36]. For the specimens from the unaffected segments, 83 unique proteins were reported. For the specimens from the affected segments, 74 unique proteins were reported. Consequently, 12 up-regulated proteins and 21 down-regulated proteins were identified in affected versus unaffected hepatic segments.

**Figure 3:**
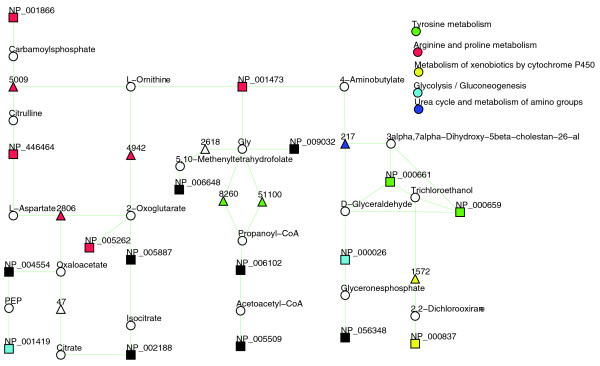
**Network model *D*_2 _of 21 down-regulated proteins in a comparison of unaffected versus affected hepatic segments **[36]. The network model *D*_2 _covers 17 proteins (*p*-value < 0.001). Proteins from the input list are indicated by rectangles, intermediate proteins by triangles, and chemical compounds by circles. The colors are used to specify KEGG canonical pathways.

For example, 17 out of 21 down-regulated proteins (unaffected versus affected hepatic segments) map to KEGG pathways. A standard enrichment analysis for the 21 down-regulated proteins found two pathways 'urea cycle' (five proteins) and 'glycolysis' (four proteins) to be enriched (Table 2). These results enable the conclusion that some characteristic metabolic pathways are violated in affected hepatic cells. Analysis with KEGG spider provides a comprehensive picture of the characteristic metabolic perturbations between normal and diseased cells. The model *D*_2_, in which proteins are connected via one intermediate protein, covers all 17 proteins (*p*-value < 0.001) that are mapped to KEGG metabolic pathways. The model *D*_2 _is presented in Figure 3. The KEGG spider model retrieves a comprehensive picture of the genetic basis of metabolic variations in comparison to standard enrichment analyses. As in the previous example, it demonstrates that deregulated genes are not independent (or split to independent pathways) and all 17 metabolism related proteins form non-interrupted (a maximum of one missing gene is allowed) network.

**Table 2:** KEGG metabolic pathways enriched in the list of 21 down-regulated proteins [36] (affected versus unaffected hepatic segments) reported by GENECODIS

Number of genes	P-value (not corrected for multiple testing)	KEGG pathway
5	4.98E-12	(KEGG) Urea cycle and metabolism of amino groups
4	7.98E-08	(KEGG) Glycolysis/gluconeogenesis

### Large scale benchmark of KEGG spider

To support the practical significance of KEGG spider, we collected dozens of recently published experimental studies that reported lists of genes/proteins in various biological contexts. We reanalyzed them using KEGG spider and demonstrated that, in most cases, the models provided by KEGG spider improve our understanding of the genetic basis of metabolism variations. These results can be found at the KEGG spider web site [37].

Of particular interest are the studies that report differentially expressed genes/proteins between normal/disease cell states or treated/untreated cell states. We selected 17 such studies, which report at least eight genes/proteins that can be mapped to KEGG metabolic pathways and analyzed these genes/proteins using KEGG spider and GENECODIS. The comparative statistics is provided in Table 3. The 'GENECODIS' column reports results provided by GENECODIS, the 'k' column reports the number of pathways found to be enriched at a *p*-value < 0.05, and the 'max' column reports the number of input genes covered by the largest pathway. As can be seen, in all cases the interpretational power of enrichment analyses was quite limited. On average, from 10% to 40% of the input genes mapped to KEGG pathways could be interpreted by one canonical pathway. As can be seen, in all cases KEGG spider provided statistically valid models.

**Table 3:** Large-scale comparison between KEGG spider and GENECODIS

		Input proteins/genes		GENECODIS		KEGG spider		
				
Paper	Table	All	**KEGG**	k	**max**	Model	*n*	*P*-value
Proteomic analysis of primary cell lines identifies protein changes present in renal cell carcinoma [40]	Table 1: proteins found to be differentially expressed between matched normal and RCC primary lines	62	23	5	10	*D*_3_	22	<0.01
Proteomic analysis of anaplastic lymphoma cell lines: identification of potential tumour markers [41]	Table 2: proteins overexpressed in FE-PD cells compared to SU-DHL-1 cells	41	13	3	3	*D*_2_	12	0.015
Differential expression profiling of human pancreatic adenocarcinoma and healthy pancreatic tissue [42]	Table 3: proteins at higher levels in normal pancreas compared to pancreatic cancer	40	12	2	5	*D*_3_	12	0.015
Proteomic search for potential diagnostic markers and therapeutic targets for ovarian clear cell adenocarcinoma [43]	Table 1: differentially expressed proteins in human ovarian cancer cells	36	17	3	4	*D*_2_	13	0.025
Quantitative proteomic analysis to discover potential diagnostic markers and therapeutic targets in human renal cell carcinoma [44]	Table 3: differentially expressed proteins in RCC patients	91	36	12	14	*D*_2_	33	<0.001
Protein profile changes in the human breast cancer cell line MCF-7 in response to SEL1L [45]	Table 4: MCF7-SEL1L differentially expressed genes identified by microarray analysis	60	9	1	4	*D*_2_	7	0.03
Protein dysregulation in mouse hippocampus polytransgenic for chromosome 21 structures in the Down syndrome critical region [46]	Table 2: list of proteins dysregulated in hippocampus of polytransgenic micea	42	14	2	5	*D*_2_	12	0.015
Differential expression of proteins in response to ceramide-mediated stress signal in colon cancer cells by 2-D gel electrophoresis and MALDI-TOF-MS [47]	Table 1: list of identified proteins on HCT116 2-DE gels	82	16	2	4	*D*_3_	15	0.02
Subcellular proteome analysis of camptothecin analogue NSC606985-treated acute myeloid leukemic cells [48]	Table 2: functional classifications of the deregulated proteins in NSC606985-induced apoptotic NB4 Cellsa	88	15	1	5	*D*_3_	15	<0.001
Proteome analysis of responses to ascochlorin in a human osteosarcoma cell line by 2-D gel electrophoresis and MALDI-TOF MS [49]	Table 2: differentially expressed proteins in ascochlorin-treated U2OS cells	87	13	3	5	*D*_2_	12	<0.001
Quantitative proteomic and genomic profiling reveals metastasis-related protein expression patterns in gastric cancer cells [50]	Table 1: summary of differentially expressed proteins and their functional classifications	227	59	11	9	*D*_3_	54	<0.001
Proteomic analysis of the resistance to aplidin in human cancer cells [51]	Table 1: differentially expressed proteins between resistant and wild-type HeLa cells identified in the membrane fraction	26	8	5	3	*D*_2_	6	0.02
Proteomic analysis of the resistance to aplidin in human cancer cells [51]	Table 2: differentially expressed proteins between resistant and wild-type HeLa cells identified in the cytosolic fraction	37	11	5	7	*D*_2_	11	0.015
Identification of specific protein markers in microdissected hepatocellular carcinoma	Table 2: identified proteins from HCC and nontumorous liver tissue by in-gel digestion and SELDI-MS	51	20	8	4	*D*_2_	17	0.015
Comparison of membrane-associated proteins in human cholangiocarcinoma and hepatocellular carcinoma cell lines [52]	Table 1: list of proteins from the membrane fraction of HuCCA-1 and HCC-S102 cell lines which show up-regulated expression	56	11	2	5	*D*_3_	11	<0.001
Contribution of laser microdissection-based technology to proteomic analysis in hepatocellular carcinoma developing on cirrhosis [53]	Table 1: proteins differentially expressed in tumorous LM-hepatocytes and total homogenates samples identified PMF	43	20	0	0	*D*_3_	18	0.04
Proteome alterations induced in human white blood cells by consumption of Brussels sprouts: results of a pilot intervention study [54]	Table 1: protein alterations induced by a controlled dietary intervention with Brussels sprouts in human primary white blood cells	44	17	2	4	*D*_2_	12	<0.05

The 'Paper' column reports the title of the paper that reported a list of differentially expressed proteins/genes related to different diseases or treated/untreated cell states. The 'Table' column reports the table number and legend from the paper. The 'Input proteins/genes' section reports the total number of proteins/genes (All) and the number (KEGG) that mapped to KEGG pathways. The 'GENECODIS' section reports results provided by GENECODIS; the 'k' column reports the number of pathways found to be enriched (*p*-value < 0.05); the 'max' column reports the number of input genes covered by the largest pathway. The 'KEGG spider' section reports results provided by KEGG spider; the 'Model' column specifies the most significant model (*D*_2 _or *D*_3_); the '*n*' column reports the number of input proteins/genes covered by the model; the *p*-value column reports significance estimated by a Monte Carlo simulation procedure.

## Conclusion

Recent advances in genomics technologies allow for the detection of genes with differential activities between various cell states. Since metabolic processes are at the heart of the cell, they are often subjected to variations in disease cell states. Complete understanding of metabolism variations can give clues to possible metabolism-related treatment of the studied cell disorders. As has been demonstrated, KEGG spider provides a comprehensive interpretation of genomics data related to metabolism variations. In addition, the KEGG spider network models incorporate not only genomics information, but also specify small molecules whose metabolism might be affected. This feature provides a link between genomics and rapidly developing high-throughput metabolomics technologies. It is obvious that experimental studies utilizing both techniques in parallel will become popular in the near future. For such studies, the interpretational models provided by KEGG spider are a useful link between genomics and metabolomics data.

We would like to point out that the idea to infer the network model from a gene list based on external knowledge is not completely new; for example, there are commercial packages available, such as Ingenuity Pathway Analysis software [38], which transforms a list of genes into a set of networks according to internal database information of gene pairwise relationships. As we already mentioned, some free online tools exist [18-21] that allow one to visualize several metabolic pathways together that are related to the input gene list. However, visual analyses of graphical representations of genes on metabolic pathways gives only an intuitive feeling that discovered genes are related. Taking into account the density of the global gene metabolic network, one must not underestimate the value of the statistical treatment. Even for randomly generated gene lists, it is possible to connect many of genes into a subnetwork through one or two intermediate partners. A beautiful looking figure may have low scientific value without statistical treatment of the presented network model.

To our knowledge not one of the currently existing tools that infer network models from gene lists provides robust statistical treatment of the inferred network models. For example, the statistical scores provided by Ingenuity Pathway Analysis do not take into account the topology of the reference network and provide statistically significant scores even for random gene lists. In contrast, KEGG spider implements a robust statistical treatment of the inferred network models, based on the topology of the global metabolic network, and provides a valid estimate of the *p*-values by a Monte Carlo simulation procedure. The *p*-values provided by KEGG spider actually reflect the probability of getting the same size network model for a random gene list.

Examples of analysis of disease-specific genes by KEGG spider suggest that the separation of metabolic reactions into canonical pathways is, to some degree, artificial. In most cases, metabolism-related genes were from several KEGG canonical pathways. However, the analysis with KEGG spider reveals that, if one considers the topology of the global gene metabolic network, these genes form a non-interrupted (a maximum of one or two genes are missing) disease-specific pathway that runs through several canonical pathways. These results also support a hypothesis that disease-specific metabolism variations in most cases are not independent, for example, deregulated genes from different pathways are linked to each other via consecutive one- or two-step metabolic reactions. The examples of analysis of disease-specific genes by KEGG spider presented in Table 3 may serve as support for this hypothesis.

Finally, we would like to summarize the power and limitations of KEGG spider. In comparison to other tools, KEGG spider provides a robust analytical framework for interpretation of gene lists in the context of a global gene metabolic network. The information of gene pairwise relationships is widely exploited (gene A is related to gene B via metabolite C) and the inferred network model is not limited to the size of one metabolic pathway. In the current form, KEGG spider computes the minimal distance between any two genes as a minimal number of steps required to get from one gene to another. A more realistic way to model distance between genes will be a weighted approach where one would consider not only the number of steps but also the impact of each step. This methodological extension can be considered as a possibility for future improvement of KEGG spider. We also would like to point out that the produced output models are limited by the available information on cell metabolism from the KEGG database.

## Materials and methods

### A global gene metabolic network

The KEGG REACTION database is a collection of chemical structure transformation patterns for substrate-product pairs (reactant pairs). We can build a global 'reaction network' (reactions are nodes, compounds are edges) by connecting with edges reactions that share the same compounds. In general, a reaction consists of multiple reactant pairs, and the one that appears on the KEGG metabolic pathway is called the main pair. To build a global reaction network, we used only compounds classified as main reaction pairs. Otherwise, many reactions will be connected only because they use or produce such compounds as H_2_O, CO_2_, and so on.

In KEGG, reactions are linked to orthologous groups of enzymes (KEGG ORTHOLOGY database) and orthologous groups are mapped to the genes (in most cases each orthologous group corresponds to ortholog genes from different genomes). Thus, reactions can be mapped to genes from a given genome, and the reaction network can be transformed into a global organism-specific gene metabolic network, where genes are nodes and compounds are edges, respectively. Some reactions are organism specific or are not annotated by an orthologous group. In this case, they are not present in the corresponding organism-specific gene network. Therefore, the resulting global gene metabolic network links by edges any two genes that are associated with reactions sharing common compounds (from the main reaction pair).

### Network inference procedure

The distance between two arbitrary genes is computed as the minimum number of consecutive steps required to get from one gene to another by working through existing paths on the global gene metabolic network. Distance 1 means that two genes are directly connected. Distance 2 means that two genes are connected via one intermediate gene, distance 3 means that two genes are connected via two intermediate genes, and so on. Given a gene list, our purpose is to infer the network model that minimizes the distance between each connected gene pair according to pairwise distances between genes.

Initially, we map genes from the input list onto the global gene metabolic network. At this point all genes from the input list are disconnected. In the first step, we connect by edges gene pairs with distance 1 and look for connected subnetworks. The subnetwork with the maximal number of genes is referred to as an inferred network model *D*_1_. We also refer to the number of genes in the maximal subnetwork as the size of the inferred model. In the second step, genes (from the input list) with distance 2 are connected by edges. The subnetwork with the maximal number of genes is inferred and is referred to as network model *D*_2_. In a similar way, network models *D*_3_, *D*_4_,..., are inferred. Models *D*_2_, *D*_3_,..., incorporate genes that are not from the input list but are added to connect input genes in the network model. We refer to these added genes as intermediate genes.

### Statistical treatment

The null hypothesis is that the input gene list has no bias in relation to the topology of the global gene metabolic network. A quality measure of the inferred network model can be its size, that is, the number of genes from the input list in the model. We have to estimate the probability to infer models with the same or bigger size from randomly generated gene lists of size *N*, where *N *is the number of input genes.

Let us assume that we have *N *genes in the input list. Using the network inference procedure described above, we infer the network models *D*_1_, *D*_2_, *D*_3_. Let us denote *S*_1_, *S*_2_, *S*_3 _to be the number of input genes in the inferred network models *D*_1_, *D*_2_, *D*_3_. The values *S*_1_, *S*_2_, *S*_3 _are used as statistics. To estimate the significance of the inferred model *D*_1_, we compare the value *S*_1 _with a distribution *R*_1*j*_. In the same way, we estimate the significance of the inferred models *D*_2_, *D*_3 _by comparing the values *S*_2_, *S*_3_with distributions *R*_2*j*_, *R*_3*j*_, respectively.

The distributions *R*_1*j*_, *R*_2*j*_, *R*_3*j *_are computed by a random simulation procedure [39]. To generate the background distributions *R*_1*j*_, *R*_2*j*_, *R*_3*j*_, we repeat the following simulation procedure *k *times. Index *j *= *1..k *specifies the random simulation. Each time the random gene list *B*_*j *_of size *N *(equal to the size of the input list) is generated. The network inference procedure described above is applied to the list *B*_*j *_and the network models *D*_1*j*_, *D*_2*j*_, *D*_3*j *_are inferred. Let us denote the number of genes from the random list *B*_*j *_in the inferred network models *D*_1*j*_, *D*_2*j*_, *D*_3*j *_as *R*_1*j*_, *R*_2*j*_, *R*_3*j*_. Thus, after repeating *k *times the simulation procedure, we get the background distribution *R*_1*j*_(*j *= *1..k*) for model *D*_1_, the background distribution *R*_2*j*_(*j *= *1..k*) for model *D*_2_and the background distribution *R*_3*j*_(*j *= *1..k*) for model *D*_3_.

To estimate the significance of the inferred network model *D*_1 _for the input gene list, the value *S*_1 _is compared to the distribution *R*_1*j*_. Let *n *be the number of values from the distribution *R*_1*j *_that are equal or greater than *S*_1_. The estimate of the *p*-value *p *of the inferred network model *D*_1 _is computed as *p *= (*n *+ 1)/*k*. In the same way, the *p*-values for models *D*_2 _and *D*_3 _are computed using values *S*_2 _and *S*_3 _and background distributions *R*_2*j *_and *R*_3*j*_. In other words, the *p*-value is estimated as a share of random simulations where the size of the inferred models for a random gene list (size *N*) are equal to or greater than the size (*S*_1_, *S*_2_, *S*_3_) of the inferred models for input gene list (size *N*).

## Abbreviations

KEGG: Kyoto Encyclopedia of Genes and Genomes.

## Authors' contributions

AAV conceived of the study and developed software, analyzed the data and drafted the manuscript. SD developed a web tool, analyzed the data and drafted the manuscript. HWM conceived of the study, and participated in its design and coordination. All the authors read and approved the final manuscript.

## Additional data files

The following additional data files are available with the online version of this paper. Additional data file 1 is a full comparison of KEGG spider to KEGG atlas.

## Supplementary Material

Additional data file 1 Full comparison of KEGG spider to KEGG atlas. Click here for file
