# Supplementary material for: KEGG spider: interpretation of genomics data in the context of the global gene metabolic network
Source: Genome Biol. 2008 Dec 18;9(12):R179. doi: 10.1186/gb-2008-9-12-r179 (PMC2646283; doi:10.1186/gb-2008-9-12-r179)
Supplement: Additional data file 1 — Full comparison of KEGG spider to KEGG atlas. [file gb-2008-9-12-r179-S1.doc]

**Supplementary Material for the paper:**

**KEGG spider: interpretation of genomics data in the context of the global gene metabolic network**

Alexey V. Antonov, Sabine Dietmann, Hans W. Mewes

As we admit in the paper, several available tools provide visualizations of a gene list in the context of the global metabolic network, however, without analytical and statistical analyses. Here, we compare KEGG spider and KEGG Atlas [23]: The 3 gene lists used for comparison are presented in Table 1,2, and 3. To reproduce our results, one just needs to copy and past gene identifiers from the corresponding column in the Table and submit them to KEGG spider [26] or KEGG atlas [55].

We want to stress that KEGG spider is an analytical tool, which implements a robust statistical framework for the analysis of gene lists in the context of the global gene metabolic network. The visualization capabilities of KEGG spider are not the major point; they are only used as a supplementary means to improve the interpretability of the analytical report. On the contrary, KEGG Atlas provides only a graphical interface with global “view-and-zoom-in” capabilities.

The advantage of KEGG spider over KEGG Atlas becomes apparent from three examples provided below. The first two examples are related to the examples described in the paper. In both cases, KEGG spider provides analytical reports that genes from the list form a non-interrupted network; and it also provides a confidence score for this event: the probability that a random gene list of the same size will have the same size non-interrupted network component. KEGG Atlas provides only a graphical representation of genes on the global metabolic network. From this output the user can get only intuitive feeling that the genes under consideration are related. Taking into account the density of the gene metabolic networks, one must not underestimate the value of the statistical treatment. The third examples aims to stress the importance of a quantitative analysis: A random gene list was generated and submitted both to KEGG spider and KEGG atlas. The KEGG atlas output was nothing different from the first two examples. It is clear that even in a random gene list some will be related just by chance. As KEGG atlas provides no quantitative analytics and does not take into account the number of input genes, the user always get a model and no evaluation of the quality of this model. On the contrary, KEGG spider clearly indicates that a submitted gene list is nothing better than random.

Tables 1 and 2 provides genes that were used in the first [35] and second [36] examples in the paper. Table 1 reports differentially expressed genes from the first example that mapped to the KEGG orthology database (not all of them are mapped to metabolic pathways). Table 2 reports differentially expressed genes from the second example that mapped to the KEGG orthology database (not all of them are mapped to metabolic pathways). Table 3 specifies the random gene list used in the third example.

In all tables, the first column specifies Entrez gene IDs, the second column specifies the mapped KEGG identifier (KO).

### KEGG Atlas, Example 1 [35]

###

### On submitting a list of KO identifiers (table 1, second column) to KEGG Atlas the user will get the output presented in the Figure 1. We want to admit that KEGG Atlas provides zoom-in capabilities, which allow for zooming different parts of the figure. As we admitted in the paper, visual analyses of graphical representation of genes on the global metabolic network give only intuitive feeling that metabolic reactions regulated by the genes are related.


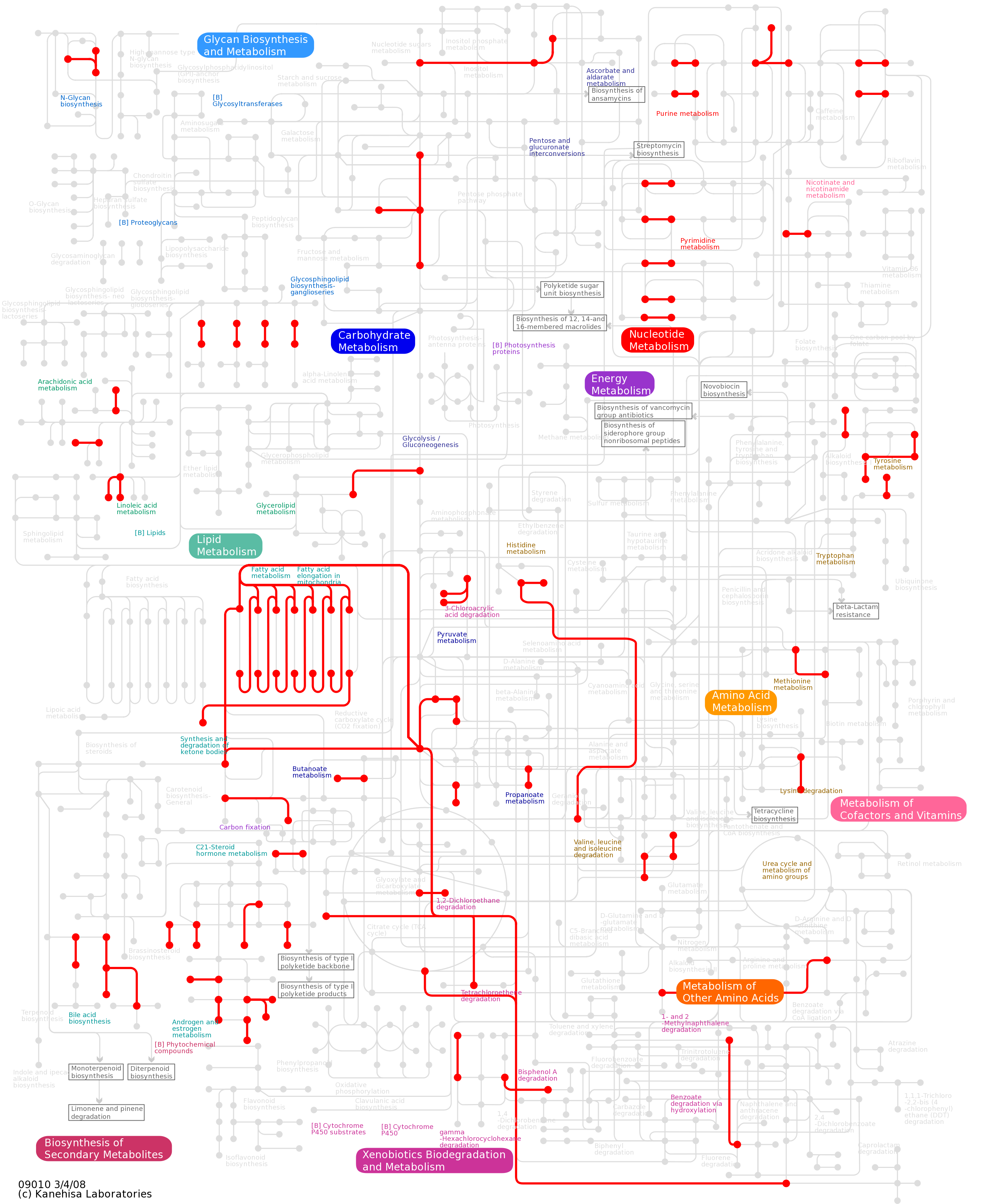


**Figure 1. The output of the KEGG Atlas (**example 1,[35]**).**

**KEGG Spider, Example 1**

On submitting a list of NCBI Entrez gene IDs (see Table 1, column 1) to KEGG spider the user will get the following analytical report:

1. 14 genes from total 28 that mapped to KEGG metabolic pathways form a non-interrupted network.
2. 24 genes form a non-interrupted network, if one missing node (distance 2) between any two input genes is allowed.
3. In both cases KEGG spider provides the confidence of both models: p-value <0.001, e.g. the chance for a random list of 28 genes (from KEGG metabolic pathways) to have the same size of non-interrupted network (model D1 and model D2) is less then 1 in 1000.

In addition, KEGG spider provides a visual output of the inferred network models presented below in Figure 2, which is more compact and concentrated only on the analysed genes.


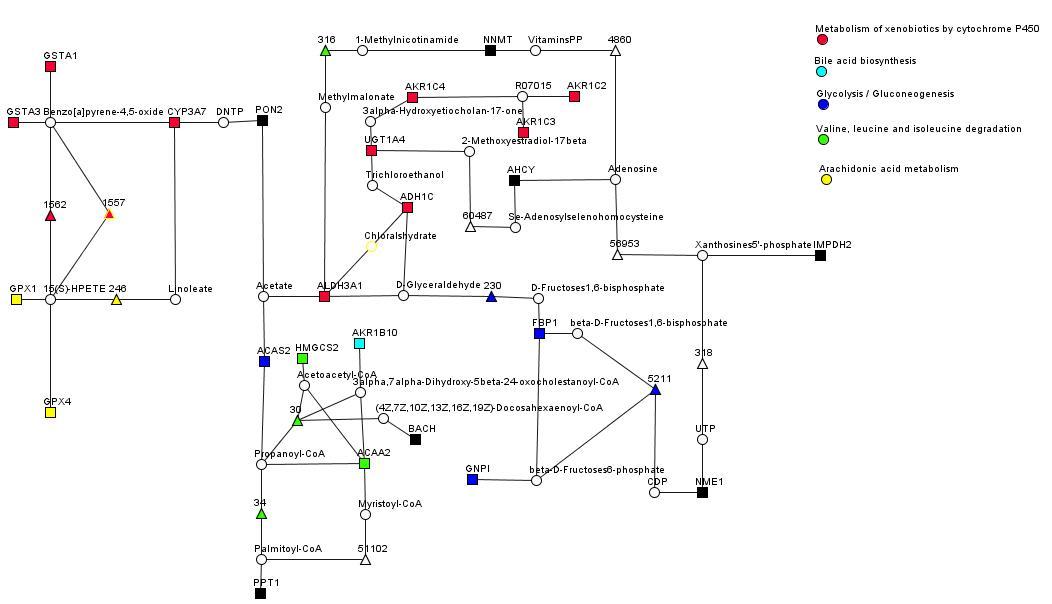


**Figure 2. Visual output of KEGG spider, example 1 [35].** Genes from the input list are presented by rectangles, intermediate genes are presented by triangles and chemical compounds are presented by circles. Different colours are used to specify different KEGG canonical pathways.

### KEGG Atlas, Example 2 [36]

###

### On submitting a list of KO identifiers (Table 2, second column) to KEGG Atlas, the user will get the output presented in the Figure 2.


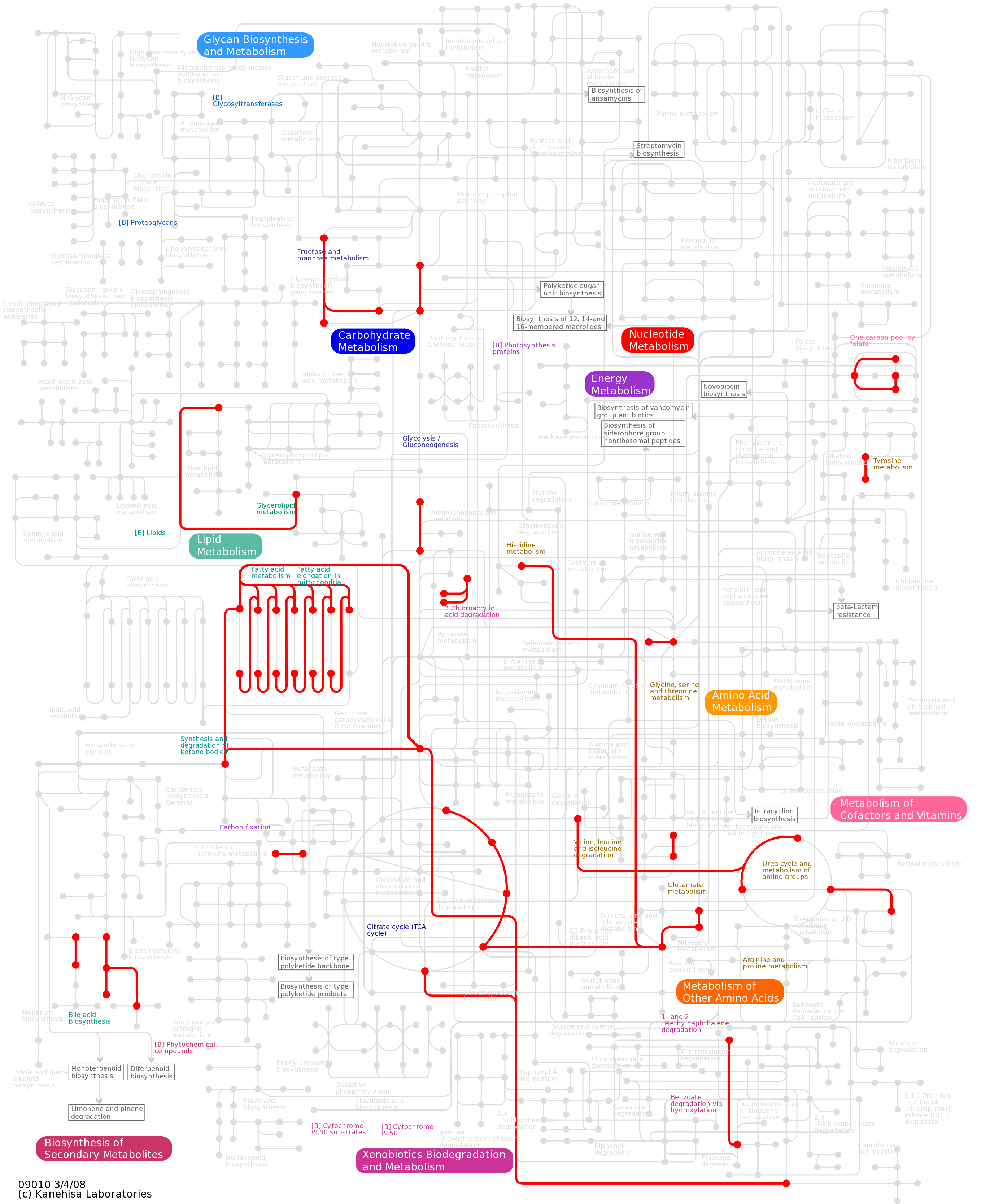


**Figure 2. The output of the KEGG Atlas (**example 2, [36]).

**KEGG Spider , Example 2** [36]**.**

On submitting a list of NCBI Entrez identifiers (Table 2, first column) to KEGG spider the user will get the following analytical report:

.

1. 16 genes (from total 17 that are mapped to KEGG metabolic pathways) form a non-interrupted network if to allow one missing node (distance 2) between any two input genes.
2. KEGG spider provides the confidence the model: p-value <0.001, e.g. the chance for a random list of 17 genes (from KEGG metabolic pathways) to have the same size of non-interrupted network (model D1 and model D2) is less then 1 in 1000.

In addition, KEGG spider provides a visual output of the inferred network model presented below in Figure 4.


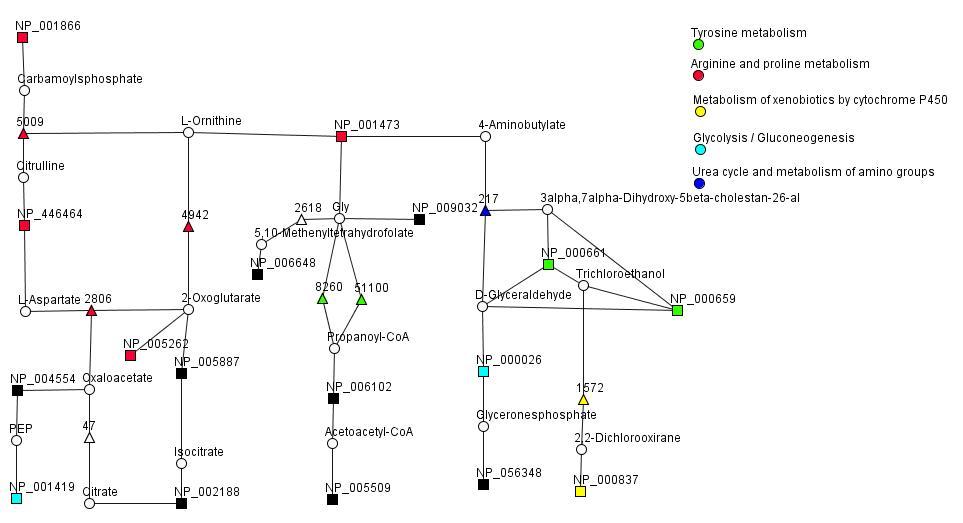


**Figure 4. Visual output of KEGG spider, example 2 [36].** Genes from the input list are presented by rectangles, intermediate genes are presented by triangles and chemical compounds are presented by circles. Different colours are used to specify different KEGG canonical pathways.

### Random list.

### On submitting of a random list of 30 KO identifiers (table 3, second column) to KEGG Atlas the user will get the output presented in Figure 5. As one can see, graphical output provided by KEGG Atlas looks not much different then the Figures 1, 3 provided for experimentally derived gene lists. It is clear that output of KEGG atlas provides no ground for making any conclusions about the quality of the gene lists used in the examples 1, 2 and 3.


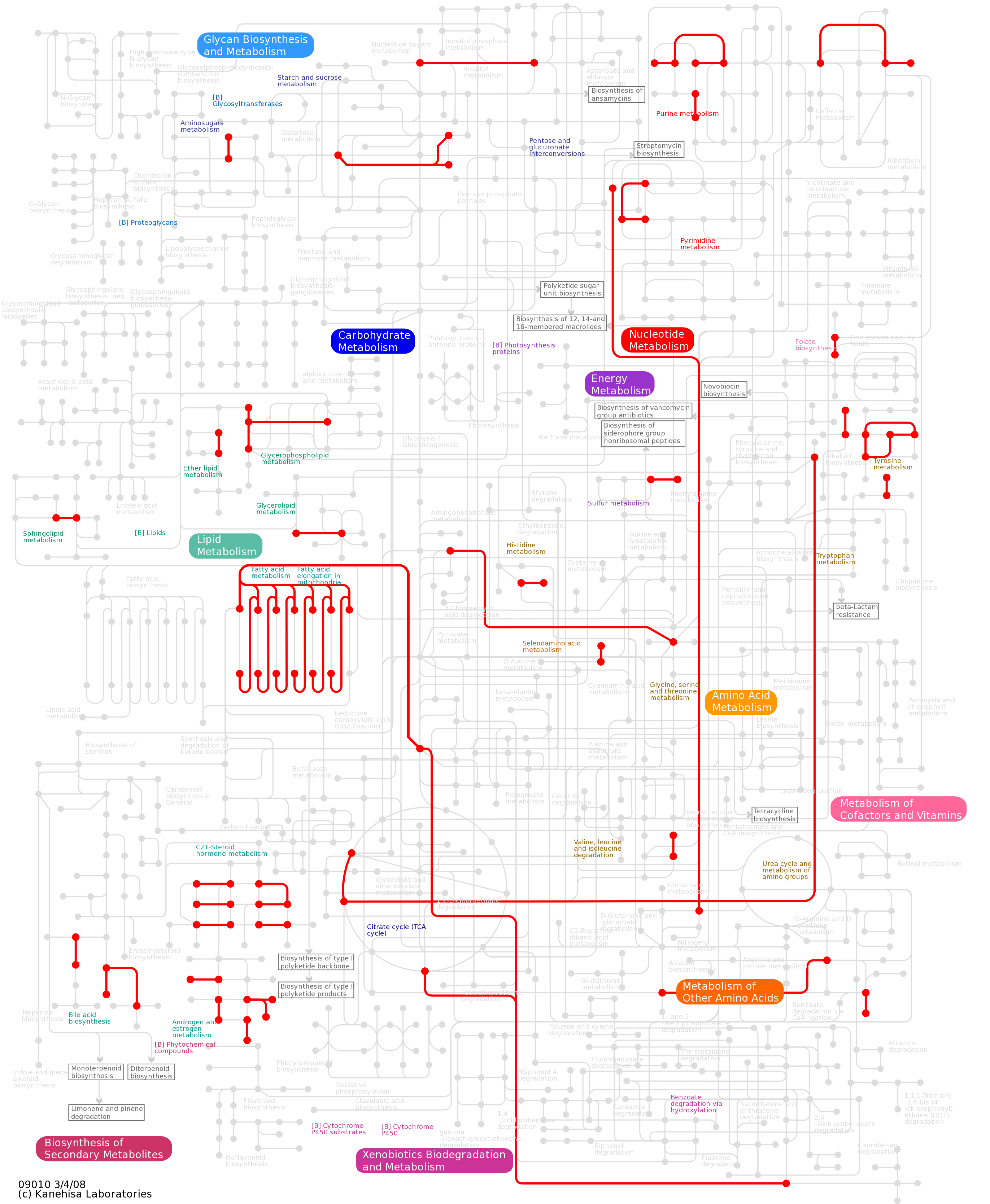


**Figure 5. The output of the KEGG Atlas for a random list of genes.**

On the contrary, the KEGG spider provides clear evidences that the considered random list of 30 gene identifiers (Table 3, first column) is nothing better than random. The significance of network models, whatever the number of missing nodes was very low, for model D_1_ p-value was ~0.25 and for model D_2_ p-value was about 0.5 (see Figure 6, screenshot of KEGG spider report).


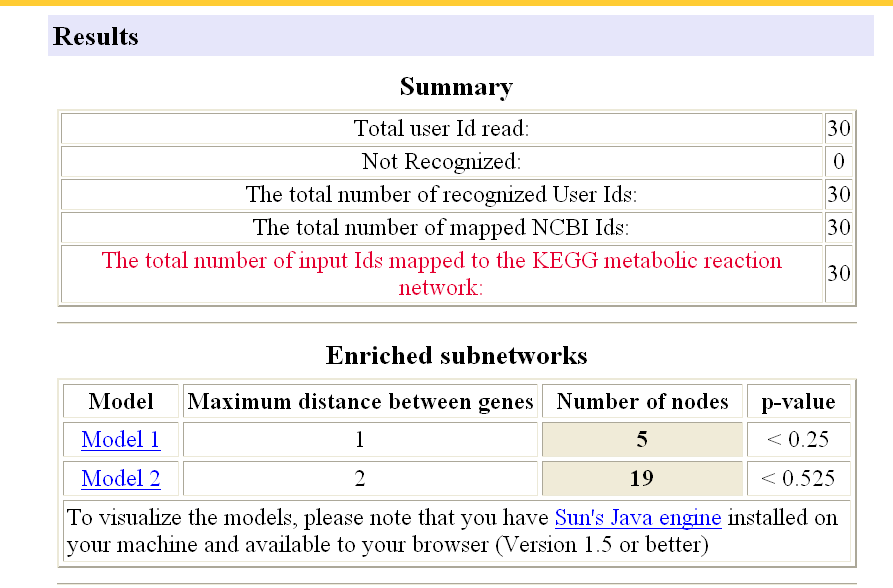


**Figure 6. The output of the KEGG Spider for a random list of genes.**

# Tables

**Table 1.** Genes commonly up- or down regulated in diffuse-type gastric cancers [35]

(Example 1, an Id mapping table, NCBI Entrez gene ID to KEGG orthology database)

| NCBI  Entrez gene ID | KEGG ortology (KO) |
| --- | --- |
| 7791 | K06273 |
| 4316 | K01397 |
| 1646 | K00089 |
| 1646 | K00212 |
| 9775 | K03257 |
| 55902 | K01895 |
| 1808 | K07528 |
| 10449 | K07508 |
| 994 | K05866 |
| 1109 | K00037 |
| 1109 | K00092 |
| 1109 | K00089 |
| 1109 | K00212 |
| 9052 | K08468 |
| 10195 | K03845 |
| 2203 | K03841 |
| 2335 | K05717 |
| 9886 | K07867 |
| 489 | K05853 |
| 727 | K03994 |
| 1281 | K06236 |
| 760 | K01672 |
| 710 | K04001 |
| 218 | K00129 |
| 218 | K00128 |
| 191 | K01251 |
| 2771 | K04630 |
| 3738 | K04876 |
| 1366 | K06087 |
| 2938 | K00799 |
| 54474 | K07604 |
| 54657 | K00699 |
| 1551 | K07424 |
| 3158 | K01641 |
| 3615 | K00088 |
| 4061 | K06846 |
| 126 | K00001 |
| 483 | K01538 |
| 1278 | K06236 |
| 2821 | K01810 |
| 10581 | K06566 |
| 114609 | K05403 |
| 1284 | K06237 |
| 3376 | K01870 |
| 1282 | K06237 |
| 2118 | K09431 |
| 2879 | K00432 |
| 10576 | K09494 |
| 6035 | K01168 |
| 3855 | K07605 |
| 8705 | K00715 |
| 5538 | K01074 |
| 3326 | K04079 |
| 1277 | K06236 |
| 991 | K03363 |
| 5445 | K01045 |
| 5445 | K01140 |
| 4830 | K00940 |
| 3248 | K00069 |
| 2289 | K09571 |
| 8237 | K01072 |
| 23428 | K03450 |
| 2168 | K08750 |
| 4837 | K00541 |
| 1508 | K01363 |
| 9518 | K05504 |
| 7850 | K04387 |
| 64499 | K01340 |
| 5591 | K06642 |
| 5465 | K07294 |
| 57016 | K00100 |
| 2940 | K00799 |
| 8644 | K00089 |
| 8644 | K00212 |
| 8644 | K04119 |
| 1358 | K01298 |
| 5225 | K01377 |
| 10392 | K08727 |
| 183 | K09821 |
| 332 | K08731 |
| 975 | K06508 |
| 2876 | K00432 |
| 11332 | K01068 |

**Table 2.** Down-regulated proteins in affected versus unaffected hepatic segments ( an Id mapping table, NCBI Entrez gene ID to KEGG ortology database)

| NCBI  Entrez gene ID | KEGG ortology (KO) |
| --- | --- |
| 127 | K00001 |
| 1373 | K01948 |
| 3158 | K01641 |
| 48 | K01681 |
| 26007 | K00863 |
| 445 | K01940 |
| 2746 | K00261 |
| 3417 | K00031 |
| 125 | K00001 |
| 2939 | K00799 |
| 1757 | K00314 |
| 10841 | K00603 |
| 10841 | K01746 |
| 3848 | K07605 |
| 10449 | K07508 |
| 5106 | K01596 |
| 3329 | K04077 |
| 229 | K01623 |
| 2023 | K01689 |
| 2628 | K00613 |

**Table 3.** A random gene list.

| NCBI  Entrez gene ID | KEGG ortology (KO) |
| --- | --- |
| 22928 | K01008 |
| 5435 | K03014 |
| 1589 | K00513 |
| 79071 | K10203 |
| 8528 | K00272 |
| 2271 | K01679 |
| 205 | K00939 |
| 1723 | K00226 |
| 56894 | K00655 |
| 8644 | K00089 |
| 8644 | K00212 |
| 8644 | K04119 |
| 1632 | K01825 |
| 1040 | K00981 |
| 5439 | K03008 |
| 3081 | K00451 |
| 4952 | K01099 |
| 8560 | K04712 |
| 5149 | K01120 |
| 8443 | K00649 |
| 10449 | K07508 |
| 4128 | K00274 |
| 79799 | K00699 |
| 10965 | K01068 |
| 2953 | K00799 |
| 1118 | K01183 |
| 57696 | K01529 |
| 8694 | K11155 |
| 166012 | K07779 |
| 1607 | K00901 |
| 7365 | K00699 |
| 6611 | K00802 |
